# Gas transport across the low-permeability containment zone of an underground nuclear explosion

**DOI:** 10.1038/s41598-020-58445-1

**Published:** 2020-01-29

**Authors:** Charles R. Carrigan, Yunwei Sun, Steven L. Hunter, David G. Ruddle, Matthew D. Simpson, Curtis M. Obi, Heather E. Huckins-Gang, Lance B. Prothro, Margaret J. Townsend

**Affiliations:** 1 Lawrence Livermore National Laboratory, Livermore, California, USA; 2 Mission Support and Test Services, LLC, Las Vegas, Nevada USA

**Keywords:** Environmental sciences, Hydrology

## Abstract

Understanding the nature of gas transport from an underground nuclear explosion (UNE) is required for evaluating the ability to detect and interpret either on-site or atmospheric signatures of noble gas radionuclides resulting from the event. We performed a pressure and chemical tracer monitoring experiment at the site of an underground nuclear test that occurred in a tunnel in Nevada to evaluate the possible modes of gas transport to the surface. The site represents a very well-contained, low gas-permeability end member for past UNEs at the Nevada National Security Site. However, there is very strong evidence that gases detected at the surface during a period of low atmospheric pressure resulted from fractures of extremely small aperture that are essentially invisible. Our analyses also suggest that gases would have easily migrated to the top of the high-permeability collapse zone following the detonation minimizing the final distance required for migration along these narrow fractures to the surface. This indicates that on-site detection of gases emanating from such low-permeability sites is feasible while standoff detection of atmospheric plumes may also be possible at local distances for sufficiently high fracture densities. Finally, our results show that gas leakage into the atmosphere also occurred directly from the tunnel portal and should be monitored in future tunnel gas sampling experiments for the purpose of better understanding relative contributions to detection of radioxenon releases via both fracture network and tunnel transport.

## Introduction

As part of the National Nuclear Security Administration’s (NNSA) program to evaluate different signatures that are characteristic of underground nuclear explosions (UNEs), we have embarked on a multi-year study of two UNE containment sites having extremely different gas transport characteristics. The first phase of the study focused on the containment site resulting from the 1989 Barnwell UNE carried out within the volcanic layering of Pahute Mesa at the Nevada National Security Site (NNSS). That UNE used a vertical emplacement design having a detonation point at 600 m depth and an explosive yield between 20 and 150 kilotons^1^. This “open” containment regime end-member was found to have a characteristic bulk gas permeability of more than 4 Darcys (1 Darcy = 1.0 × 10^−12^ m^2^) as shown in Table 1 of ^2^ and Table 1 of ^3^.

More recently the Barnwell end-member has been used to simulate atmospheric signatures resulting from the seepage of gases from the detonation cavity or chimney to the surface and into the atmosphere^4^. Using coupled simulations of subsurface and atmospheric transport, it was found that current levels of sensitivity applicable to atmospheric radioxenon analyses^5,6^ would allow the detection of atmospheric plumes at distances of more than one thousand kilometers assuming only the occurrence of late-time, noble gas seepage from a 5-kt UNE emplaced according to an accepted depth-to-yield relationship.

In this paper we evaluate the gas transport characteristics of a much lower bulk-permeability, gas containment regime. Additionally, the method for deploying the nuclear device differs from the vertical emplacement design of Barnwell. A series of access tunnels (e.g., the P-tunnel complex) was mined into the volcanic layers of Aqueduct Mesa at NNSS. Drifts were then mined into the sidewalls of the tunnels where an explosive device could be emplaced and followed by back-filling or plugging the drift with grout back to the tunnel before detonation. Among four UNEs performed in the P-tunnel complex, our research focused on the gas transport characteristics of the Disko Elm (DE) event which occurred in September 1989 having a yield less than 20 kt^7^.

At the P-tunnel DE site, we performed field experiments relevant to estimating gas leakage parameters by monitoring the propagation of atmospheric pressure fluctuations into the DE chimney. Additionally, we injected a Freon gas tracer into the DE chimney with the objective of determining where leakage might occur within and above the complex before escaping into the atmosphere. We found very strong evidence during gas sampling near surface-ground-zero (SGZ) on Aqueduct Mesa that a prolonged period of barometric low pressure, which occurred several weeks after injection, caused migration of tracer gas from the DE chimney to the surface, which our simulations suggest could be the result of narrow fractures with apertures formed by very uneven surfaces containing many asperities. We also detected significant leakage of tracer from the chimney and into the tunnel complex almost immediately following its injection into the chimney.

The field tracer injection/sampling experiments were followed by computer modeling based on the NUFT hydrologic transport program^8^. NUFT was used to perform multi-parameter variational studies to estimate relevant leakage parameters using the atmospheric pressure data. We also used NUFT to obtain an independent evaluation of the DE chimney permeability using the Freon injection-pressure and flow rate data. The significance of tracer detections on the surface of the mesa and in the tunnel will be considered in the following sections. We will also include a discussion of the end-member containment regimes we have considered to date and implications of the level of containment for standoff detection and characterization of noble gas isotopic signatures.

**Figure 1:**
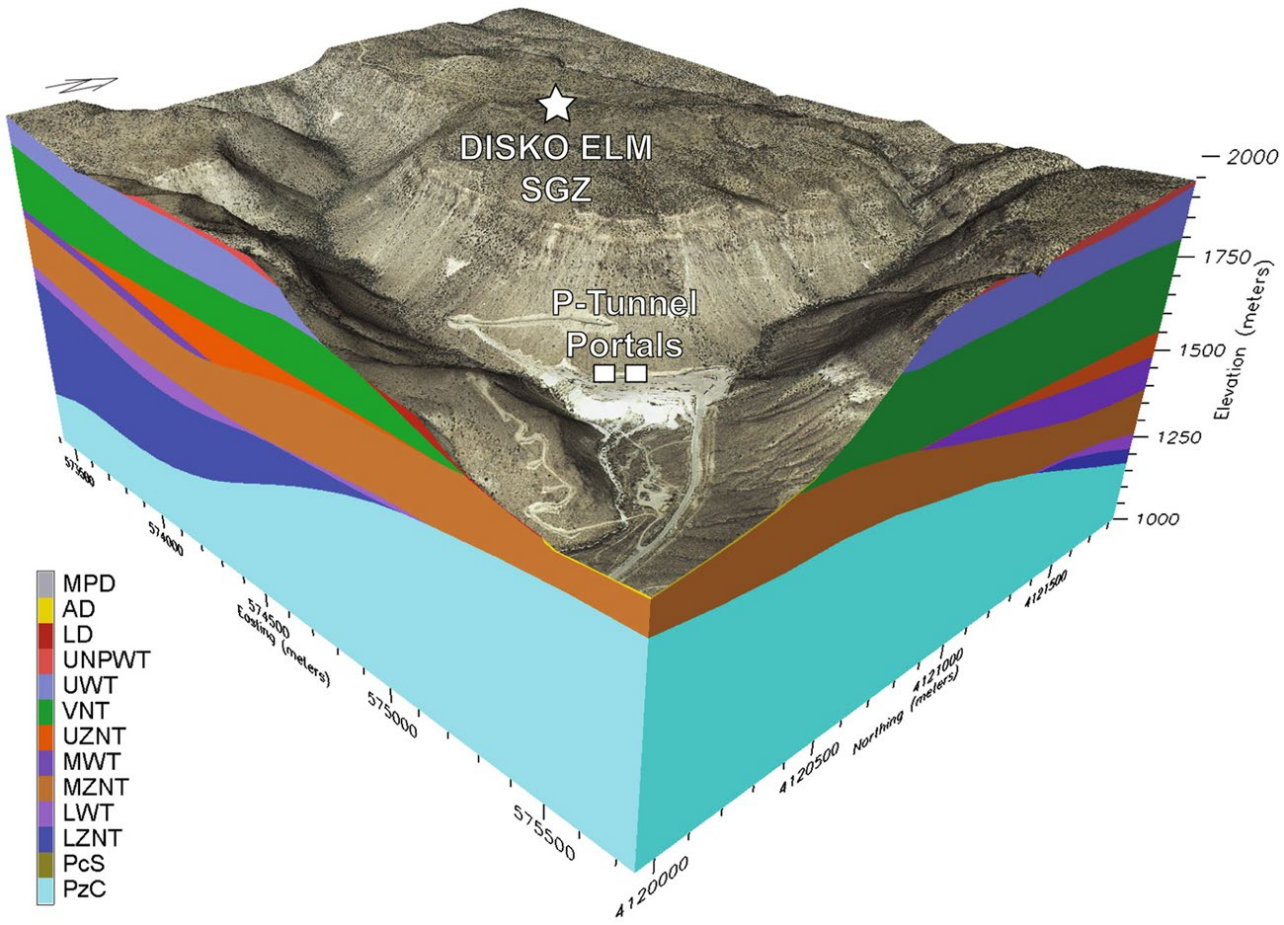
The Disko Elm nuclear test occurred in the P-tunnel complex within Aqueduct Mesa at the Nevada National Security Site (NNSS). The Portal at the base of the mesa accesses a tunnel at 5520 feet or 1725 m altitude. Surface-ground-zero (SGZ) or the point directly above the detonation is at 6377 feet or 1993 m altitude. The detonation point is at the tunnel level or 1725 m altitude yielding an equivalent depth of burial beneath the mesa of 268 m, which places it near the top of the UZNT or zeolitized non-welded or bedded tuff geologic formation (orange layer in figure). Image generated using EarthVision 10, Dynamic Graphics Inc., Alameda, CA^14^.

**Figure 2:**
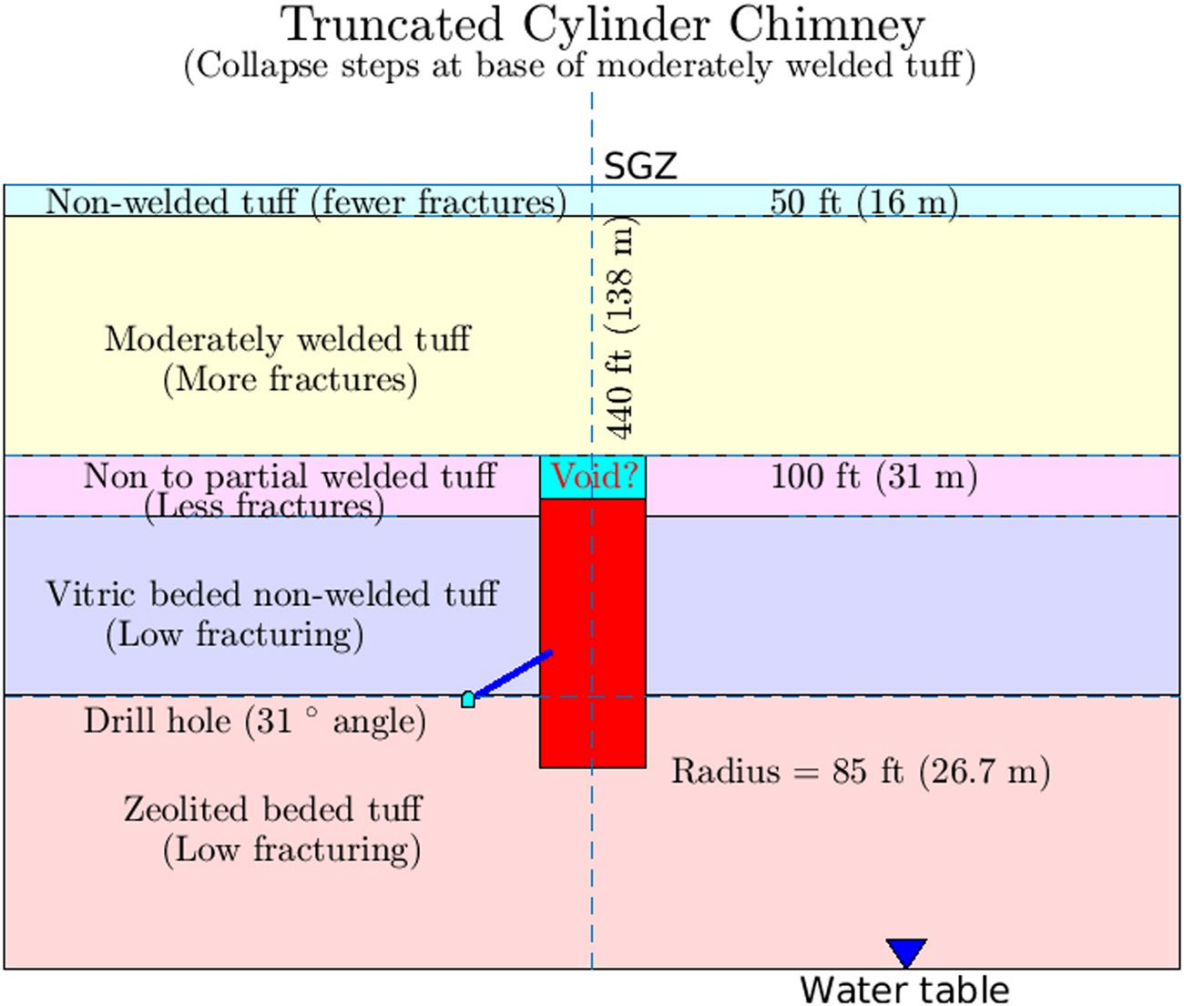
The relationship of the angled tracer-injection borehole to the detonation or working point is shown along with the distribution of bulk permeability assumed in this study. Both cavity injection pressure and barometric pressure are measured through this angled borehole terminating in the rubblized material resulting from the collapsed roof. We define characteristic bulk permeabilities for the rubble material (*P*_*r*_), the void at the top (*P*_*v*_ = infinity) and the containment regime (*P*_*c*_).

The device was detonated near the top of the zeolitized non-welded tuff (UZNT) geologic unit (Fig. 1, orange layer) at a depth of 268 m beneath the top of Aqueduct Mesa. Between the detonation point and SGZ there are several other volcanic formations exhibiting varying levels of strength and fracture characteristics. Additional details of the geologic cross section are provided in Fig. 2. As the post-detonation, cavity-forming pressure decreased below levels capable of supporting lithostatic loading above the cavity, collapse of its roof occurred. The preferred model (Fig. 2) for the chimney structure resulting from the upward migration of the collapse has it extending upward 141 m through two geologic units to the base of a stronger, moderately welded tuff (UWT) unit, which resisted further collapse. (Note: If the cavity reaches the surface a crater is formed, but in this case the welded layer of volcanic tuff is believed to have halted the upward migration of the void during the collapse.) This unit is also more highly fractured than the ones it overlies making it less of a barrier to gas transport from the chimney and into the surrounding zone of containment. The radius of the chimney is likely to vary with height. The angled borehole from the tunnel into the chimney intersected its boundary at a radial distance from the detonation point of 26.7 m, which we will assume to be the radius of a simplified cylindrical model of the DE chimney.

The interior of the zone of collapse or chimney usually consists of a zone of melt at the bottom of the chimney resulting from the heat of detonation overlain by rubblized material from the collapse of the cavity roof. The ability of the collapsed material or rubble to maintain some level of structural integrity allowing very high-permeability fracture pathways for gas to exist has usually been assumed permitting chimney models to have a uniform noble gas distribution (e.g.,^2,4^). However, the low strength of the non-welded tuff in this case may allow elimination of many gas paths as a result of the crushing or compaction of the collapsed tuff during the upward cavity-migration or stoping process. For the purpose of this study, we have assumed a two-permeability cavity having a zone of lower permeability underlying a void (with high permeability) that has migrated upward from the detonation point to the base of the moderately welded tuff unit as shown in Fig. 2.

## Barometric Pressure Observations

LLNL has developed a gas sampling and environmental parameter logging system called the Subsurface Gas Smart Sampler (SGSS). The sampling systems are ruggedized for field use and operate indefinitely on either electrical mains power in the tunnel complex or on batteries charged by solar panels. On Aqueduct Mesa two systems extracted daily soil-gas samples (0.5-liter) from beneath tarps while continuously monitoring barometric pressure and radon levels at SGZ. Four other SGSS units were positioned at locations in the tunnel complex to allow sampling of tunnel air after the injection of Freon tracer in addition to monitoring atmospheric pressure fluctuations in the chimney via the injection borehole and in the tunnel complex.

**Figure 3:**
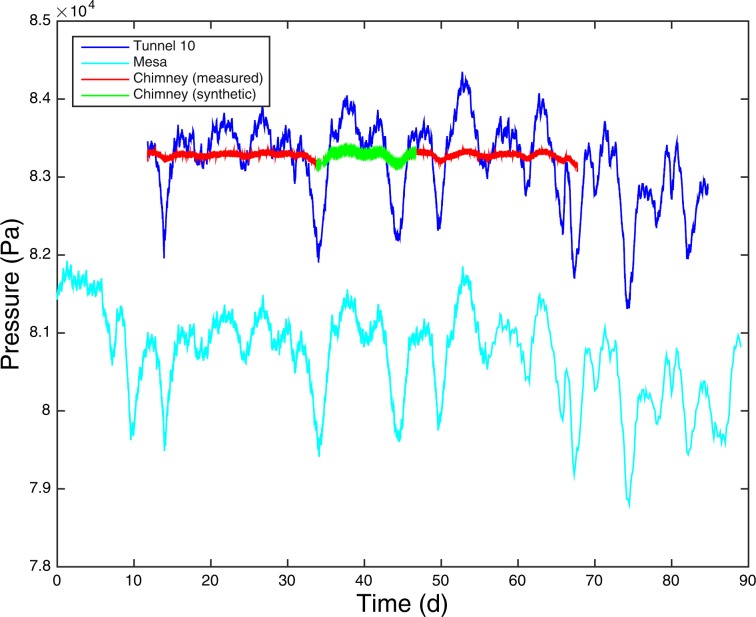
Illustrated are fluctuations in barometric pressure monitored in the tunnel (i.e., tunnel barometer, dark blue line) and at SGZ on the mesa surface (light blue line). The pressure offset due to the elevation difference between the tunnel and SGZ (~268 m) is about 25 millibars (mb). When this offset is eliminated with the different vertical scales in the figure, the pressure signals at SGZ (light blue line) and in the tunnel (dark blue) essentially overlap each other. The red line is the cavity pressure history which fluctuates very little (~1 mb) compared to the other measurements for most of the pressure record. For part of the record, the cavity pressure signal appears to track the tunnel pressure history because the cavity pressure-measurement line was disconnected from the borehole prior to injection to prevent damage to the pressure sensor and then left to record tunnel pressure variations. The green line is the cavity pressure synthesized using the measured atmospheric pressure fluctuations (light blue line).

We used the barometric pressure in Non-Isothermal, Unsaturated Flow and Transport (NUFT) simulations^8,9^ to estimate characteristic values of containment-zone bulk permeability, fracture aperture, fracture frequency and matrix permeability, which is discussed in a subsequent section. However, Fig. 3 shows several interesting details concerning the different pressure measurements. All the measurements, except in the cavity, overlap each other when altitude offsets are taken into account for the SGZ measurements. Even the partially sealed drift (RE-3) mined back to within approximately 6 meters of the edge of the chimney rubble zone tracks the tunnel and SGZ fluctuations closely. This very good tracking of pressure fluctuations indicates that the mined-back drift is better connected to the main tunnel complex than expected. Secondly, the range of cavity-pressure fluctuations is only 7–8% of the range of atmospheric fluctuations that drive the cavity-pressure changes. By comparison, the Barnwell chimney pressure variations are more than 30% of the range of atmospheric pressure fluctuations (Fig. 1 of ^2^). The more highly attenuated amplitude of the chimney signal clearly indicates that the atmosphere-to-monitoring-borehole connection is much weaker (lower permeability) than in the Barnwell containment regime. However, is this mainly a result of the DE containment regime or the potentially low permeability of the zone of collapse above the monitoring borehole? We investigate this using tracer-injection-pressure monitoring data.

## Freon Injection Observations

Prior to injection, sampling of tunnel gases was performed for several days to determine what gases might already be present. One of the tracer candidates, Freon 13B1, was not detected in any samples. In addition to its very low to nonexistent background, the tracer is essentially inert, like a noble gas, and has a detection level of approximately 0.1 parts-per-billion-by volume (ppbv). Its molecular weight (149) is comparable to the radioxenon isotopes of interest allowing it to have a similar gas diffusivity to radioxenon.

During a period of almost 4 hours, we injected 63 kg of Freon 13B1 into the Disko Elm chimney along a 39 m annular region formed between two concentric tubes grouted into the borehole connecting the access tunnel to a region in the collapse zone located above the detonation or working point (Fig. 2). The Freon was co-injected with ambient air into the rubblized chimney material through the borehole for approximately 220 minutes at a rate of 6.1 m^3^ per minute. The injection rate was held constant during this period except for a very brief stoppage of the pump to determine the pressure drop, about 8 mb, along the length of the 39 m annular injection path (Fig. 3). Following this period, the flow rate was reduced to 4.46 m^3^ min^−1^ for about 20 minutes before terminating the injection. The injection-pressure history data, shown in the section of Analysis of Injection-Pressure Data, are a best fit curve since the pressure data set available to us was incomplete with a gap in the middle during the 220-minute period.

An important question concerning any estimate of the bulk leakage parameters of the zone of containment is how estimating these parameters by monitoring barometric-pressure-driven fluctuations might be affected when the borehole used for tracking pressure changes terminates in the rubble-filled collapsed zone. We used the injection-pressure history to estimate the role that this chimney-collapse zone of rubble plays in influencing the overall bulk leakage parameters obtained independently from the propagation of atmospheric pressure fluctuations. Two models of the distribution of gas permeability in the chimney were used to determine a best fit to the injection-pressure data, which will be considered in a subsequent section.

**Figure 4:**
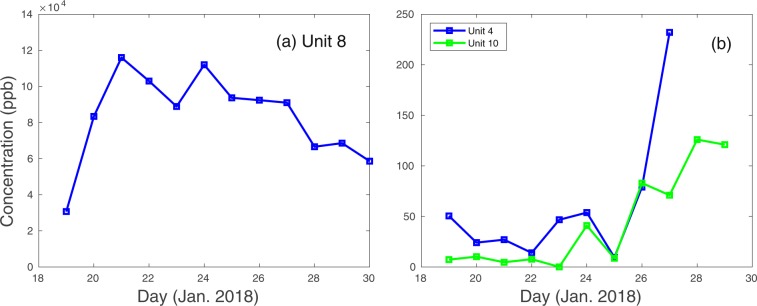
Freon signals versus time in the re-entry drift (**a**), in the access tunnel alcove outside the re-entry drift door (**b**, blue line) and in the access tunnel at about 200 m from the alcove by the ventilation intake (**b**, green line). The tracer histories indicate that the chimney apparently leaked directly into the re-entry drift producing a high concentration of tracer diluted less than 1/50 of the injection concentration. Gases from the drift then diffused into the access tunnel and were transported down the tunnel to the ventilation intake where they could be potentially exhausted out the tunnel portal.

The injection was completed just before 4 pm on 18 January 2018. Gas samples were taken periodically by three smart samplers in the injection borehole access tunnel as well as in the post-detonation re-entry drift via a port in the metal door to the drift and in an alcove adjacent to the re-entry drift door. The samples were then sent for analysis using a specially tuned gas chromatograph coupled to an electron-capture detector (ECD). The analyses indicated that tracer was present in the re-entry drift within 24 hours of completing the injection. Figure 4 shows how Freon levels changed with time at the three sampling locations in (1) the re-entry drift, (2) the alcove next to the re-entry drift door and (3) the access tunnel 200 m distant. It is highly likely that tracer from the Disko Elm chimney first entered the P-tunnel complex through the rear wall of the re-entry drift about 6 m from the edge of the chimney. The concentration of tracer gradually increased in the re-entry drift until it was detected beyond the drift door in the adjacent alcove and finally in the tunnel. The gradual migration of gases from the chimney into the tunnel has significant implications for the standoff detection of a tunnel-emplaced UNE especially when the containment geology may have a very low bulk permeability.

**Figure 5:**
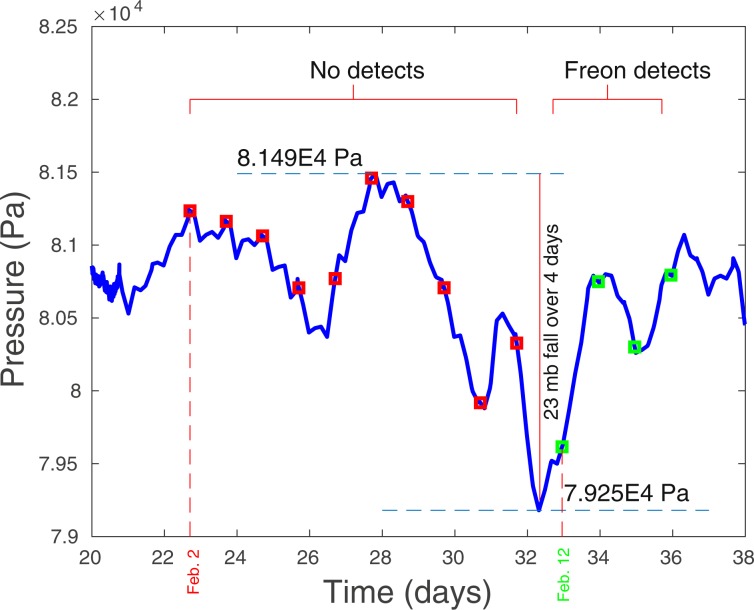
A long period of falling barometric pressure at SGZ coincides with the termination of 10 consecutive no detects during 2–11 February 2018 by four sequential Freon detections starting on 12 February. Barometric pressure fell 22 mb during a 4-day period. It is estimated that the altitude-corrected pressure in the chimney, which was monitored during this time, should have produced a flow toward the surface whenever the barometric pressure fell below approximately 808 mb at SGZ. All 4 detects occurred when the atmospheric pressure was either below or about 808 mb when gases would be flowing from the Disko Elm chimney. This observation further supports the fracture origin of the detections since no such requirement exists for releases to occur from the tunnel.

On the surface of Aqueduct Mesa at SGZ, two smart samplers were sampling periodically during a prolonged drop in atmospheric pressure of almost 22 mb over 4 days, which followed the injection by about three weeks. Between the middle and end of the period of reduced barometric pressure, gas samples taken from beneath one of the sampled tarps yielded 4 consecutive detections of Freon between 12–15 February as shown in Fig. 5. The detections of Freon terminated a period of 10 consecutive “no detects” obtained in the preceding 10-day period. It should be noted that the other smart sampler, which obtained samples every other day beneath a tarp near to the first tarp, captured no tracer during the same period. This is significant as it indicates that the source of tracer captured beneath the first tarp was highly localized, such as resulting from a fracture. Any atmospheric plume that might have originated from the tunnel portal about a kilometer distant would be expected to produce detections under both tarps at SGZ.

## Analysis of Barometric Pressure Monitoring Data

We used the NUFT simulator of multiphase flow and reactive transport in a porous medium to perform a best fit variational study of four leakage-related parameters to match the observed chimney borehole pressure fluctuations (Fig. 3) driven by surface barometric pressure variations. Variation of the five parameters required for the dual permeability models (DKM) used in NUFT (bulk permeability, fracture aperture, fracture frequency and matrix permeability) to obtain the best fit to the borehole pressure fluctuations involved doing 1000 simulations yielding the best-fit leakage parameters in Table 1. Table 1 also characterizes the top ten best fits to provide an indication of the range of parameter variations that occurred. The smaller the range of the 5-parameter variations suggests the increasing stability of this approach to obtaining the leakage parameters.

**Table 1:** Using a NUFT model to simulate the propagation of pressure fluctuations between the surface of Aqueduct Mesa and the monitoring borehole embedded in the Disko Elm collapse chimney, a five-parameter variation study was performed to obtain a best fit of the pressure-fluctuation history to observations.

Parameter	Unit	Minimum	Maximum	Mean of top 10	Best
Fracture aperture	[m]	1.0000 × 10^−4^	1.0000 × 10^−2^	1.2442 × 10^−3^	8.4258 × 10^−4^
Fracture frequency	[−]	0.5000	10.000	1.2324	1.0379
Fracture permeability	[m^2^]	1.0000 × 10^−14^	1.0000 × 10^−9^	4.9911 × 10^−9^	6.9158 × 10^−10^
Matrix permeability	[m^2^]	1.0000 × 10^−18^	1.0000 × 10^−15^	9.1720 × 10^−18^	1.0014 × 10^−17^
Bulk permeability	[m^2^]	2.6655 × 10^−18^	1.3801 × 10^−11^	5.1549 × 10^−13^	4.5269 × 10^−13^

To obtain the best fit simulation yielding a bulk permeability of the containment zone of 0.45 Darcys (4.5 × 10^−13^ m^2^) required performing 1000 simulations. This value is about 1/10 of that obtained for the Barnwell site on Pahute Mesa. In addition to the best fits obtained for the five parameters, the maximum, minimum and means of the best 10 fits are also provided. The best-fit parameter set produced excellent agreement between the simulated and observed chimney-pressure fluctuation over the 20-day history that was considered.

The value of approximately 0.45 Darcys obtained for the bulk permeability is strikingly low indicating that the borehole penetrating the chimney is not well connected to the surface regarding the transport of gases to the surface especially compared to the Barnwell test site having an estimated permeability exceeding 4 Darcys^2^. How the contribution of the collapsed chimney material affects this low value of permeability is considered in the next section.

## Analysis of Injection-Pressure Data

Injection pressure as a function of time as measured at the tunnel-end of the injection path depends on the (1) injected flow rate, (2) resistance to turbulent flow along the injection pathway, (3) permeability of the chimney or rubble in the zone of collapse and (4) the bulk permeability of the zone of containment outside of the chimney. For a constant rate of injection, pressure increases asymptotically in time as gas flows into the surrounding chimney rubble as well as into the zone of containment beyond the chimney. If continued for a sufficiently long time, pressure will eventually reach a constant value. When this occurs, the rate of injection is entirely offset by the loss of gas into the zone of containment external to the chimney.

Assuming a single-permeability model for the rubble zone filling the chimney (Fig. 2), we performed a multi-parameter variational analysis to obtain a best-fit match of the actual injection pressure history to the model of Fig. 2. The dynamic model describes pressure response at the end of the borehole for a given flux of gas injected at that location. 3600 2-D planar simulations were performed in the process of varying containment-zone formation permeability, chimney permeability, vertical containment-zone fracture permeability, horizontal containment-zone fracture permeability and tunnel permeability where the tunnel is treated as a separate barometric pressure source. Additionally, a Sobol’-sensitivity study^10^ was performed to rank order the variables by their level of impact on the pressure-history model. Table 2 lists the, best-fit values of the permeability parameter ranges assumed for each variable along with the Sobol’ rank ordering of the importance of each varied parameter for obtaining the final fit. We find that containment-zone leakage properties and other parameters of Table 2 play little role compared to the bulk chimney permeability in influencing the fit of the model to the data based on the Sobol’ sensitivity ranking. The best fit for the single-permeability model collapse zone is about 100 Darcys (e.g., 1.0 × 10^−10^ m^2^), which is a very high permeability and is presumably a result of extensive fracturing within the rubblized material in the chimney.

**Table 2:** Observed and modelled injection histories were matched using a multi-parameter variational approach.

Permeability in	Sobol’ TSI	Minimum	Maximum	Calibrated
		(log_10_ m^2^)	(log_10_ m^2^)	(m^2^)
Matrix	3.4766 × 10^−6^	−18	−15	1.2534 × 10^−16^
Vertical fracture	2.2988 × 10^−6^	−14	−11	1.7094 × 10^−13^
Horizontal fracture	5.7597 × 10^−7^	−14	−11	6.3941 × 10^−12^
Tunnel	2.0250 × 10^−6^	−10	−8	2.7357 × 10^−9^
Chimney	1.0870	−11	−9	1.0148 × 10^−10^

The single-material model involved the 5 parameters listed in the table while the two-material model included a separate zone of permeability centered on the end of the borehole. Sobol’ indices for the single- and double-material studies indicate that only the chimney material permeability significantly influenced the fit of matching of the model with the observations (Sobol’ index is unity). The Sobol’ indices and best-fit values are very similar for both single- and two-material/zone models. Only the single-material/zone values are shown here.

**Figure 6:**
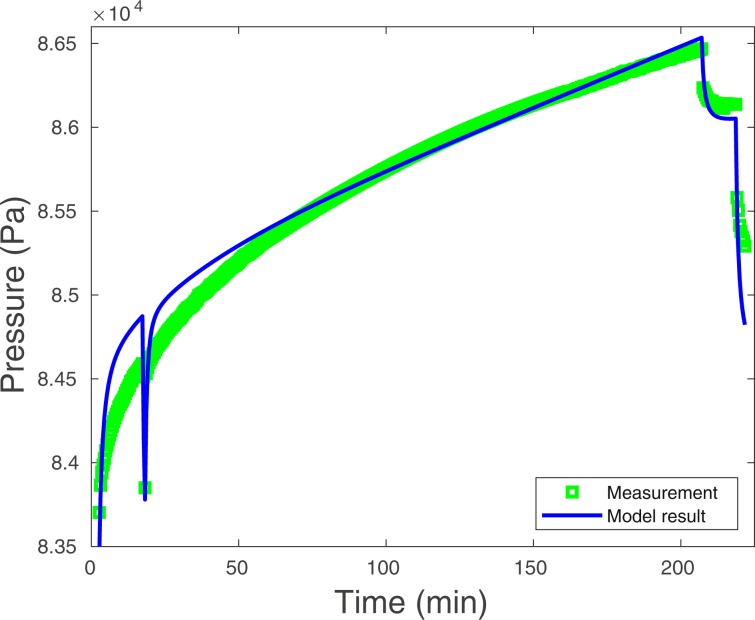
The green line is the pressure history for co-injection of the Freon-air mixture at an initial rate of 6.1 m^3^ min^−1^. After slightly more than 200 minutes or 3.6 hours, the injection flow rate was decreased to 4.6 m^3^ min^−1^ creating the rapid drop in pressure at the top right-side of the plot. This rate was maintained only briefly before injection was terminated at about 240 minutes. The match to this data for a one-permeability model representing the chimney material is given by the blue line. Both measurement and model result lines also exhibit a pressure drop out between 10~20 minutes after injection was started, which occurred when pumping was briefly halted to determine the effective static pressure drop along the length of the injection pathway (~8 mb). The agreement between measurement and single-permeability models is approximate.

We found the fit of the best single-permeability material or collapse-zone model to the pressure history data in Fig. 6 to be reasonable, given the level of uncertainty in chimney size and other parameters, but not ideal. Adding more structure to the permeability regime in the chimney was considered as a way to improve the fit, but it was found that adding an additional zone of permeability surrounding the borehole did extremely little to improve the fit between the model and observed pressure-history curves. The lack of perfect agreement between the two curves is not a great concern as it is clear other variables not considered, such as chimney size, shape and bulking factor (i.e., the ratio of void volume to chimney volume) may play a role in improving the match. Using a similar pressure-history matching approach, Peterson *et al*.^11^ found comparable order-of-magnitude estimates for the permeability of the chimney collapse material from the Ming Blade UNE which occurred in Rainier Mesa. We achieved our main objective in performing this analysis by obtaining an order-of-magnitude estimate of the bulk permeability of the chimney collapse material. Given the 100-Darcy estimated permeability of the chimney collapse zone, it can be assumed that the much lower bulk permeability (~0.45 Darcys) determined by propagation of barometric pressure fluctuations to the borehole monitoring point is entirely due to the zone of containment surrounding the chimney collapse zone and not the collapsed material itself, which is characterized by a permeability orders of magnitude higher.

## Freon Tracer Analyses

Based on barometric pressure observations, the tunnel complex within Aqueduct Mesa is characterized by a bulk fracture permeability much lower than that of the high-permeability end-member Barnwell UNE site on Pahute Mesa at the Nevada National Security Site (NNSS). However, a low bulk permeability does not rule out the possibility that a set of sparse fractures exists over SGZ, which serve as pathways for chimney gas transport to the surface (Fig. 2 of^12^). The apparent very high estimated permeability of the collapse material in the chimney suggests that chimney gases may readily move upward to the top of the chimney, which according to our preferred geologic model is at the base of a mechanically strong, but somewhat fractured, upper partially welded tuff (UWT) formation about 140 m below the surface (Fig. 2). Furthermore, post-detonation damage models of UNEs support the existence of enhanced, spall-produced fracture permeability at and just below the surface^12^. We now consider the possibility that the weak pressurization of the chimney during injection (averaging 15 mb) for about 220 minutes and the subsequent barometric pumping of gases, subject to the recorded barometric pressure history, can explain the observed arrivals and concentration levels of Freon tracer at SGZ following a long barometric low that occurred several weeks after the injection (Fig. 5).

**Figure 7:**
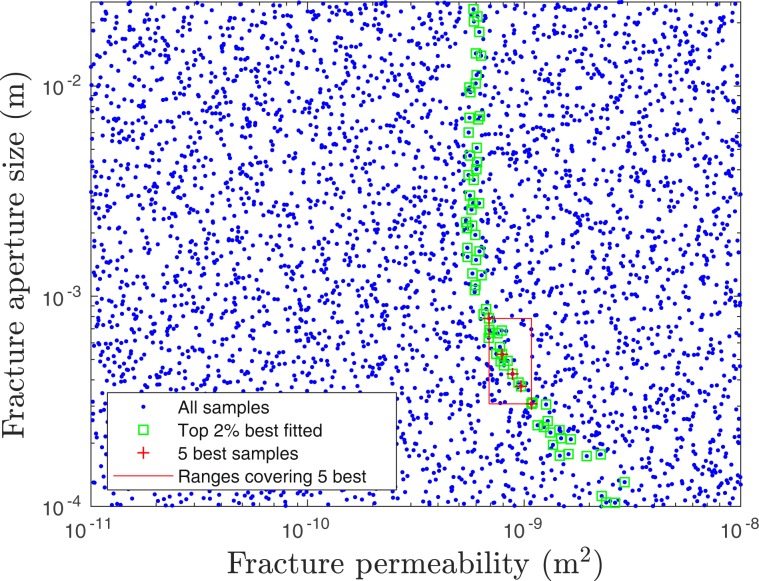
Fracture aperture size and fracture material permeability combinations selected in a two-parameter variational study are plotted. The study was performed to determine the single-fracture gas transport simulations with the best fits to the observed Freon arrivals and concentrations at SGZ on Aqueduct Mesa. The parameter combinations for the top 5 solutions fall within the red box giving the ranges of those parameters. The best fit is for the combination: aperture = 5.29 × 10^−4^ m, fracture material permeability = 791 × 10^−12^ m^2^. The green circles highlight parameter combinations that fall within the top 2% of solutions.

Our NUFT simulations of combined injection-pressure-driven and barometric transport of the Freon tracer are based on the geometry of Fig. 2. The model assumes a single fracture extending from the top of the chimney to the surface over the uppermost 140 m of the containment zone. A time-dependent Freon source term, based on the injection data, is added in the injection period. Beyond that, the concentration field including the chimney and void is evolved by mixing resulting from barometric pumping. An initial Sobol’ sensitivity analysis evaluated the effect of a number of model variables influencing transport along a fracture. Fracture aperture of the single vertical fracture and permeability of the material in the fracture were found to be the most important parameters for determining the nature of tracer transport. A two-parameter variational study was then performed for 3600 combinations of these two parameters to identify the combination giving the best fit to the first arrival of Freon and also the concentrations of the tracer as measured at the same time on 4 consecutive days. As illustrated in Fig. 7, a relatively large region in the 2-D parameter space was explored involving ranges of 3 orders of magnitude for fracture permeability (1.0 × 10^−8^ ~ 1.0 × 10^−11^ m^2^) and more than 2 orders of magnitude (1.0 × 10^−4^ ~ 2.5 × 10^−2^ m) for fracture aperture. The goal of this search over such large parameter ranges was to look for multiple best-fit-parameter combinations, although only one was ultimately identified falling inside the box in Fig. 7, which identifies the ranges containing the five best fits to the observations. Outside the box, green-black symbols show parameter combinations falling within the top 2% of fits. It is interesting that no good match can be found unless the material in the fracture has a permeability of approximately 600–700 × 10^−12^ m^2^ or higher. Only when the permeability of material in the fracture is around this value do good fits (top 2%) become possible and the nearly vertical distribution of the top 2% means that any fracture between 1 mm and 2.5 cm yields a comparable fit of tracer concentration history. Only when the fracture aperture falls below about 1 mm must the permeability of the fracture material increase further to maintain a solution in the top 2% of simulations. The top 5 best fits (red box) show the permeability and aperture ranges as [6.89 × 10^−10^ 1.08 × 10^−9^] m^2^ and [0.308 0.784] mm, respectively.

**Figure 8:**
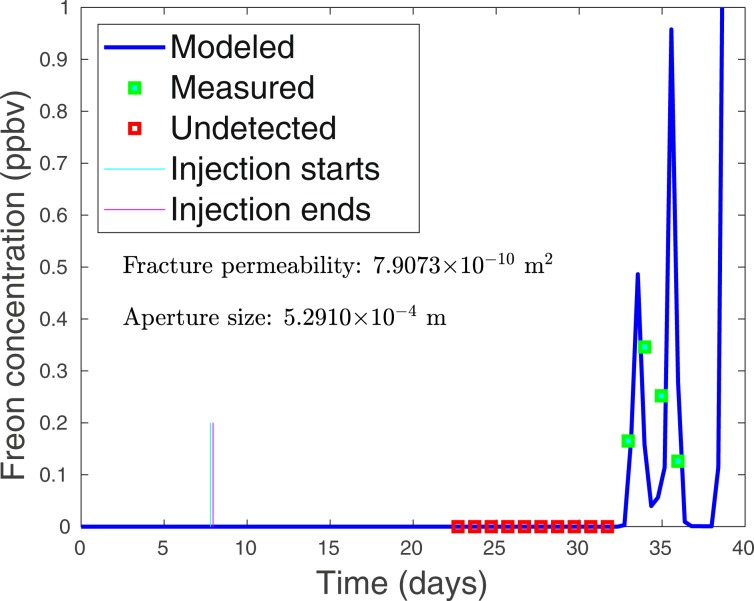
Comparison of the best-fit single-fracture barometric transport model with actual soil gas observations at SGZ provided by an LLNL tarp-and-sample system. The first 10 daily samples did not produce detections (open red squares), while the last 4 daily samples were detections (filled green squares). The concentration history simulation (blue line) essentially matches both the first arrival of the Freon and the subsequent concentration levels measured during 4 consecutive days. Note that time (horizontal axis) is measured from the start of simulation (vertical arrow) which began about a week before the actual Freon tracer injection to initialize the simulation. The 3.6-hour Freon injection ended on 18 February 2018 (vertical mark on time axis) between 7 and 8 days after the start of the simulation. Time elapsed between Freon injection and its apparent arrival at the surface is approximately 24.3 days.

Comparison of the simulated Freon-tracer history assuming the best-fit parameter combination (aperture = 5.29 × 10^−4^ m, fracture material permeability = 791 × 10^−12^ m^2^) with the data obtained from soil-gas sampling at SGZ is shown in Fig. 8. The extremely high level of agreement between the simulation and observations was unexpected and rather remarkable considering that the model appears to match not only the time of tracer arrival at SGZ but also the concentrations of all four samples containing Freon, each obtained a day apart. We conclude that the physical model for transport by a subsurface fracture, which is subject to the observed injection and barometric pressure conditions, appears very well suited to explaining these particular observations of Freon concentrations and arrival times. That a fracture is responsible for the Freon detections obtained at SGZ is further supported by the observation that detections only occurred when atmospheric pressures at SGZ fell below cavity pressures (altitude corrected) allowing tracer to migrate toward the surface. A release from the tunnel about a kilometer distant does not have this requirement. Finally, tracer was detected beneath only one of two tarps at SGZ which is indicative of a highly localized source, that is, a fracture. An atmospheric plume of gases should be detected beneath both tarps if atmospheric infiltration had been significant and a plume containing Freon originating from the tunnel portal was present.

## Discussion and Conclusions

We have used barometric and gas-injection pressure histories and a gas-tracer release to calibrate models for gas containment and release from the collapse chimney formed by the 1989 Disko Elm UNE within the P-tunnel complex of Aqueduct Mesa at NNSS. The results presented here contribute to understanding the nature of the source term or types and amounts of radioactivity released to the environment following an underground nuclear explosion. This has value for predicting the detectability of nuclear explosions by on-site and standoff radionuclide monitoring systems as well as for interpreting the characteristics of those explosions from the monitoring observations.

From monitoring of barometric pressure in the tunnel complex, it appears that (1) the DE re-entry drift is well-connected to the main access tunnel, given that there is little difference in pressure histories between the tunnel and re-entry drift. From matching the pressure history in the DE cavity-collapse structure or chimney driven by barometric pressure fluctuations on the surface and in the tunnel, we obtained (2) a bulk permeability of about 4.5 × 10^−13^ m^2^ which is very much toward the low end for bulk permeabilities of UNE containment zones. The amplitude of pressure fluctuations in the cavity is only 7–8% of the fluctuations at the surface which is only a fraction of the amplitude of the fluctuation observed in the Barnwell chimney^2^. By matching the injection-pressure history for a constant injection rate of gas into the chimney, we found that (3) the collapse zone rubble appears to have a high permeability of about 100 × 10^−12^ m^2^ suggesting that injected gases should be able to flow relatively freely in the chimney during the Freon tracer injection. Further, (4) the low bulk permeability cannot be due to loss of permeability in the collapse zone but must be a result of relatively sparse fracturing in the outer containment zone.

Injection and monitoring of Freon 13B1 tracer provides additional information about connection of the DE chimney to both the tunnel complex and surface. We found only a day after the injection that (5) significant levels of tracer had leaked into the DE re-entry drift having an end wall only about 6 m from the estimated boundary of the chimney collapse zone. Monitoring in the adjacent access tunnel indicated that (6) tracer in the sealed re-entry drift also migrated into the tunnel complex and would be eventually exhausted out the tunnel portal. Monitoring at SGZ beneath a deployed tarp yielded some surprising results (7) during a prolonged period of barometric low pressure about 3 weeks after the tracer injection when 4 detections were obtained on successive days. Fracture-transport simulations were able to provide an excellent match to the timing of the tracer arrival as well as all 4 of the measured concentrations of tracer in the samples assuming an effective fracture aperture of about 0.5 mm and a permeability of about 800 Darcys for the fracture-filling material. The estimated permeability is lower than that calculated using an idealized model for fracture apertures based on the cubic law^13^. Such a narrow, material-filled fracture would be extremely difficult or impossible to detect by any means other than localizing the soil-gas flux using a tarp-and-sample system such as that deployed in this study. It should also be emphasized that gas transport across the subsurface is not just limited to very narrow and long fractures extending from the detonation point since transport can occur by fractures emanating from the top of the very high-permeability chimney, effectively shortening by about 50% the distance of transport by fractures external to the chimney.

While geologic considerations alone may suggest the Aqueduct Mesa test site provides excellent containment of gases in the subsurface, our study supports the view that detectable gas loss via fracture transport within Aqueduct Mesa can occur following an underground nuclear explosion. The time scale of tracer arrival at the surface was appropriate for detections of most radioxenons of current interest by soil-gas sampling during on-site monitoring as well as by regional deployments of atmospheric samplers. Future tunnel studies should include both soil-gas and atmospheric sampling involving different released tracers to better understand relative contributions of these different modes of gas transport to the total gas released to the atmosphere from an underground nuclear explosion.
